# Blue LEDs get the Nobel Prize while Red LEDs are poised to save lives

**DOI:** 10.3389/fphys.2014.00443

**Published:** 2014-11-14

**Authors:** Basil S. Karam, Fadi G. Akar

**Affiliations:** The Cardiovascular Institute, Icahn School of Medicine at Mount SinaiNew York, NY, USA

**Keywords:** nitric oxide, ischemia-reperfusion injury, mitochondria, diabetes, cardioprotection

Ischemic heart disease is a major public health epidemic and a leading cause of morbidity and mortality worldwide (Hausenloy et al., 2012; Ferdinandy et al., 2014). Ischemic injury predisposes to myocardial infarction, heart failure, arrhythmias, and sudden cardiac death. Prompt restoration of oxygenated blood flow to the ischemic myocardium (i.e., reperfusion) is required for preventing irreversible cell damage and death. Unfortunately, restoration of blood flow, in itself, results in additional cardiac damage, known as reperfusion injury. Such oxidative damage, which is mediated by bursts of reactive oxygen species (ROS), is more severe when reperfusion therapy is delayed. Indeed, necrotic cell death as a consequence of ROS overproduction can paradoxically exacerbate the extent of myocardial infarction. Concomitantly, reperfusion-mediated cytosolic calcium overload and redox imbalance promote mechanoelectrical dysfunction and arrhythmias.

In recent years, mitochondria have emerged as central mediators of cell death and survival pathways (O'Rourke et al., 2005). On the one hand, opening of energy-dissipating mitochondrial channels that destabilize the mitochondrial membrane potential, such as the permeability transition pore (PTP) and the inner membrane anion channel (IMAC), result in myocardial infarction (Hausenloy et al., 2012) and arrhythmias (Akar et al., 2005), respectively. On the other hand, the seminal discovery of intrinsic cardioprotective pathways that stem from a mitochondrial origin has provided hope for combatting ischemia-reperfusion injury along with its pathological manifestations (impaired contractile recovery, arrhythmias, and myocardial infarction) (Murry et al., 1986). In particular, the proven efficacy of ischemic pre- and post-conditioning protocols in limiting the damage imposed by the index ischemic event has provided researchers with an effective tool for uncovering endogenous cardioprotective signaling pathways, with the promise of identifying molecular targets that can be manipulated pharmacologically (Ferdinandy et al., 2014).

Prominent amongst such targets are ATP-sensitive potassium channels in the mitochondrial membrane (mK_ATP_) which are tightly regulated by PKC signaling. Although the molecular identity of these channels has eluded discovery for many years, recent work by the O'Rourke laboratory convincingly points to ROMK as a viable candidate (Foster et al., 2012). Nonetheless mK_ATP_ activation by diazoxide is cardioprotective against ischemia-reperfusion injury. Another key target is the PTP whose opening represents a terminal event that causes necrotic cell death. Indeed, Hausenloy and others have shown that PTP inhibition using cyclosporine-A (CsA) effectively limits the extent of myocardial infarction (Hausenloy et al., 2012). Whether CsA protects or exacerbates post-ischemic electrical dysfunction, however, remains a matter of debate. This issue may be complicated by PKC-dependent cross-talk between the PTP and mK_ATP_ channels which we recently examined (Xie et al., 2014). Moreover, post-ischemic arrhythmias can be suppressed by stabilizing the mitochondrial membrane potential using antagonists of the peripheral benzodiazepine receptor which modulates IMAC (Akar et al., 2005). The efficacy of this strategy in limiting infarct size, however, has not been systematically tested. Finally, volatile anesthetics have also been shown to reduce reperfusion injury likely by targeting mitochondrial pathways (Agarwal et al., 2014). Because pharmacological therapies for reperfusion injury have proven difficult, novel approaches for this epidemic are much needed.

In this issue of the journal, Keszler et al. (2014) focused on a highly innovative non-pharmacological strategy. Specifically, they were able to harness the power of near-infrared (NIR) light-emitting diodes (LEDs) to liberate nitric oxide (NO) in a manner that exerted a potent cardioprotective effect. The findings of Keszler et al. (2014) are exciting on several grounds. Not only did these authors expand our understanding of the mechanism by which NIR elicits cardioprotection, they convincingly documented its utility in the setting of diabetes mellitus. This achievement cannot be overstated given the failure of most other cardioprotective strategies, including ischemic conditioning, in this setting.

## “NO” liberation by light

NIR light has been used to protect neurons from methanol toxicity, stimulate angiogenesis, heal chemotherapy-induced mucositis, and reduce myocardial infarct size through NO-dependent signaling. The beneficial role of NO is documented by studies in which its inhibition was found to abrogate the cardioprotective effects of ischemic preconditioning. Moreover, several agents known to increase NO bioavailability (for example, phosphodiesterase inhibitors, glycerol trinitrate, and nicorandil) are all potent activators of cardioprotective signaling. Of note, NO triggers mK_ATP_ channel activation through a PKG-cGMP dependent pathway, whose protective effects are abolished by PKG inhibitors or NO scavengers (Costa et al., 2008). Moreover, NO reduces mitochondrial ROS levels and oxidative stress during IR injury by trapping superoxide and eliciting conformational changes that promote S-nitrosation of Complex I of the electron transport chain (Wink et al., 1993; Paolocci et al., 2001; Chouchani et al., 2013). As such, NO markedly attenuates ROS-mediated toxicity while elevated ROS levels act to suppress basal and agonist-induced NO release (Wink et al., 1993; Paolocci et al., 2001).

Nitric Oxide Synthases (NOS), the major enzymes that produce NO, are upregulated in response to pre-conditioning stimuli. Since NOS are functionally downregulated in the context of diabetes mellitus, the cardioprotective signaling pathways that are elicited by NO are severely compromised in this setting. To circumvent this important limitation, various groups have developed a clever strategy for liberating NO directly from heme-containing proteins using NIR light. The utility of this strategy in diabetes, however, remained largely unknown—at least until now.

In an elegant Research Topic (http://journal.frontiersin.org/ResearchTopic/1809) hosted by Aon and colleagues, Keszler et al. (2014) demonstrated that NIR-mediated cardioprotection, which is likely to be NO-dependent (Lohr et al., 2009), was surprisingly NOS-independent. Treatment of hearts with the NOS inhibitor L-NAME did not significantly alter the extent of protection as the reduction in infarct size remained virtually unchanged. Likewise, the infarct-sparing effects of NIR were neither abolished in endothelial NOS deficient mice nor in a well-established model of type-2 diabetes mellitus (*db/db* mice).

The exciting findings of Keszler et al. (2014) should spur investigators to examine the potential cardioprotective efficacy of NIR as a tool for remote preconditioning. If NIR is indeed effective even when applied to remote areas and/or organs, its clinical applicability and translatability would be markedly enhanced. Given the short half-life and high reactivity of NO, the maximum allowable distance between the site of NIR application and the infarct location should be carefully determined in future studies. Finally, since NO signaling modulates numerous cellular targets, including a host of sarcolemmal ion channels and calcium regulatory proteins, it will be critical to investigate the effects of NIR on electrophysiological properties and excitation-contraction coupling. As elegantly highlighted in this Research Topic, NIR is promising in its ability to treat diabetic hearts, for which classically cardioprotective therapies have failed. Indeed, the findings of Keszler et al. (2014) break new grounds in our effort to manage diabetic patients who are at high risk of ischemia-related complications.

### Conflict of interest statement

The authors declare that the research was conducted in the absence of any commercial or financial relationships that could be construed as a potential conflict of interest.
